# Inflammation in the avian spleen: timing is everything

**DOI:** 10.1186/1471-2199-11-104

**Published:** 2010-12-31

**Authors:** Kallur S Naidu, Louis W Morgan, Michael J Bailey

**Affiliations:** 1 From The Center for Biological Clocks Research, Department of Poultry Science, Texas A&M University, College Station, TX 77843-2472, USA

## Abstract

**Background:**

The synchrony of an organism with both its external and internal environment is critical to well-being and survival. As a result, organisms display daily cycles of physiology and behavior termed circadian rhythms. At the cellular level, circadian rhythms originate via interlocked autoregulatory feedback loops consisting of circadian clock genes and their proteins. These regulatory loops provide the molecular framework that enables the intracellular circadian timing system necessary to generate and maintain subsequent 24 hr rhythms. In the present study we examine the daily control of circadian clock genes and regulation of the inflammatory response by the circadian clock in the spleen.

**Results:**

Our results reveal that circadian clock genes as well as proinflammatory cytokines, including Tnfά and IL-1β, display rhythmic oscillations of mRNA abundance over a 24 hr cycle. LPS-induced systemic inflammation applied at midday vs. midnight reveals a differential response of proinflammatory cytokine induction in the spleen, suggesting a daily rhythm of inflammation. Exogenous melatonin administration at midday prior to LPS stimulation conveys pleiotropic effects, enhancing and repressing inflammatory cytokines, indicating melatonin functions as both a pro- and anti-inflammatory molecule in the spleen.

**Conclusion:**

In summary, a daily oscillation of circadian clock genes and inflammatory cytokines as well as the ability of melatonin to function as a daily mediator of inflammation provides valuable information to aid in deciphering how the circadian timing system regulates immune function at the molecular level. However, further research is needed to clarify the precise mechanisms by which the circadian clock and melatonin have an impact upon daily immune functions in the periphery.

## Background

As the planet rotates on its axis, organisms on earth are exposed to a myriad of environmental cues, including varying daily light intensity, temperature, and solar radiation [1,2]. An endogenous timing system, collectively referred to as the biological or circadian clock, has evolved permitting organisms to match the earth's rotation, resulting in 24 hr circadian rhythms of behavior and physiology [3,4]. The term circadian indicates that, under free-running conditions, the period length (τ) of behavior and physiological functions is circa one day (*circa *about, *dies*, day). Hallmark properties for describing circadian rhythms are: 1) the rhythm must be self-sustained and persist in the absence of a light: dark (LD) cycle or other exogenous time signal (Zeitgeber); 2) the rhythm persists with a period of approximately 24 hrs; 3) the rhythm can be phase-shifted by environmental perturbations, e.g., by light; 4) the rhythm can be synchronized, or entrained, by external time cues such as the LD cycle; and 5) the rhythm exhibits temperature compensation (TC), meaning that the free-running period is approximately the same at different but constant temperatures [5].

Circadian rhythms are a pervasive property of most multicellular organisms, most eukaryotic microorganisms. and at least some prokaryotic taxa [6]. Although the study of unicellular organisms points to the cellular mechanisms responsible for generating circadian rhythms, the master circadian pacemaker in higher organisms is located in cells of specific structures of the organism. This includes regions of the brain (optic lobe) in insects; the eyes in certain invertebrates and vertebrates; and the pineal gland of some nonmammalian vertebrates [7-9]. In mammals, the circadian clock resides in the suprachiasmatic nucleus (SCN), which is located at the base of the brain in the anterior hypothalamus.

The master pacemaker function of the SCN was initially demonstrated using lesioning studies in rodents that revealed a disruption and abolishment of endocrine and behavioral circadian rhythms following SCN ablation [10]. Furthermore, transplantation studies indicated that SCN from donor animals provided to SCN lesioned animals restored most circadian rhythms [10]. Hence the SCN was long believed to be the master circadian structure responsible for generating ~24 hr rhythms of physiology and behavior in mammals. However, we now know that peripheral tissues and cells contain oscillators similar to those of the brain, termed peripheral oscillators [3]. For example, researchers have discovered that circadian rhythms persist in isolated lungs, livers, and other tissues *in vitro*, in the absence of SCN control [11]. These types of studies have led to a progressive shift in the traditional perception of circadian hierarchy, which is now regarded as an integrated timing system involving peripheral oscillators in tissues and cells throughout the body whose activities are not wholly dependent upon SCN stimulation. Rather, the SCN participates as a synchronizer of peripheral tissue oscillations [12].

It has been known for some time that circadian rhythms are genetically determined [2]. Circadian traits such as free-running periods and phase-angle (Ψ) to entraining light cycles can be selected for, and mutations for these traits have been identified, leading to the isolation and characterization of the molecular clock components [13,14]. In the last 20 years, the mRNA and protein products of the so called "clock genes" have been shown to oscillate with a genotype-specific period, and their protein products have been shown to interact *in vitro *to form the molecular circadian clock [15-17]. Comprised of interlocking transcriptional and translation feedback loops, clock genes are categorized into groups of "positive elements" and "negative elements". The positive elements Clock and Bmal1 are transcribed, translated, and then dimerize to reenter the nucleus. There, the CLOCK/BMAL1 dimer stimulates transcription via binding of genes containing an E-box motif. The E-box containing genes are the "negative elements" period1 (*per1*), period2 (*per2*), period3 (*per3*), cryptochrome1 (*cry1*) and cryptochrome2 (*cry2*). The negative elements are then transcribed, translated and form oligomers that reenter the nucleus where they inhibit the actions of the positive elements *clock *and *bmal1*. This cycle takes ~24 hrs to complete and provides an intricate molecular framework that enables the intracellular circadian timing system.

Since the discovery of the first clock gene several researchers have identified orthologs of these clock genes in several organisms [18-24]. In addition, several authors [3,19,25-27] have used microarray studies to demonstrate that many aspects of localized tissue physiology are regulated in a daily manner. These transcriptional profiling data suggest that rhythmic circadian clock gene expression may play an important role in the generation of peripheral tissue rhythms. However, important unanswered questions remain about the molecular involvement, regulation, and necessity of circadian clock genes in peripheral tissues for generating and maintaining physiological rhythms.

Recent studies involving ablation of the *per2 *gene offer clues as to the molecular basis for circadian gene necessity in the daily regulation of physiological functions such as immunity in the periphery. In one such study, *per2 *mutants lacked daily rhythms of IFN-γ (interferon gamma) in the spleen [28]. This study provides novel functional evidence for circadian gene expression in the daily regulation of immune tissue physiology and highlights the need for further examination to discern circadian-immune regulatory pathways. Understanding how disruptions of circadian timing mechanisms lead to dysfunctions of the immune response is potentially very important in understanding the progression of several inflammatory disorders and disease states. The aim of this research was to better understand the daily regulation of immunological function by the circadian clock through determining the expression pattern of circadian clock genes and the regulation of the inflammatory response over a 24 hr cycle in the spleen.

## Results

### Daily rhythms in the spleen

**Figure 1:**
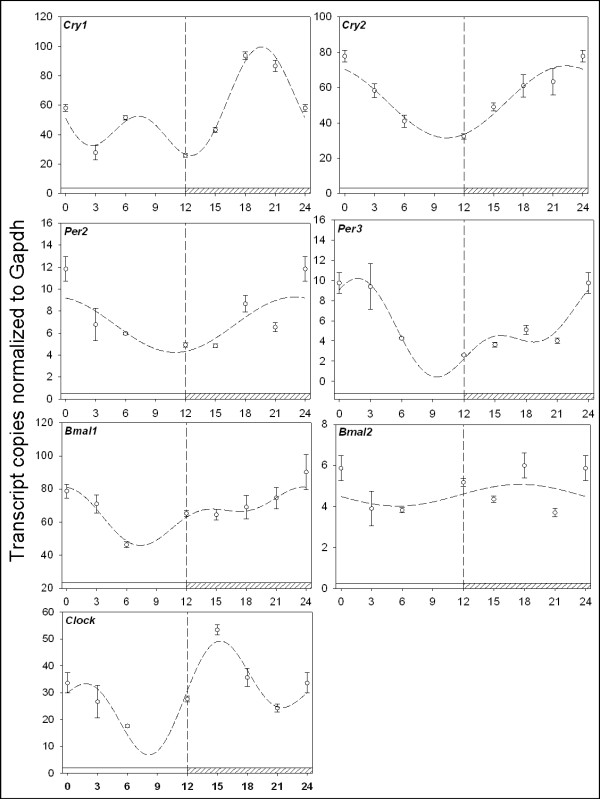
**Quantitative RTPCR analysis of circadian clock gene expression in the spleen**. Plotted open circles represent the mean ± SEM in each experimental group. The dashed line represents the fitted plot of cosinor analysis utilizing linear harmonic regression. Values are represented as the number of transcript copies/1000 Gapdh transcripts. Abscissa labels indicate ZT time values every 3 hrs under LD 12:12 conditions; ZT0 = lights on; ZT12 = lights off. Open bar on the bottom of the x-axis indicates the light period, while crosshatched indicates darkness. The *cry *and *per *genes harbor daily oscillations ~2-5 fold in amplitude with higher abundances occurring during the late night for *cry1 *(p_ANOVA _< .001; p_cosinor _= .009), *cry2 *(p_ANOVA _= .003; p_cosinor _< .001)*, per2 *(p_ANOVA _< .001; p_cosinor _= .005), and *per3 *(p_ANOVA _< .001; p_cosinor _= .008). *Clock *and the *bmals*, also express a daily pattern of rhythmicity in the spleen. Clock mRNA attained maximal abundance during the early night (p_ANOVA _< .001; p_cosinor _< .001) while *bmal1 *is highest during the late night to early day period (p_ANOVA _= .007; p_cosinor _= .01). *Bmal2 *showed increased expression during the nighttime (p_ANOVA _= .01; p_cosinor _= .03), however it was not as robust as other clock genes nor in excess of a 2-fold rhythm.

**Figure 2:**
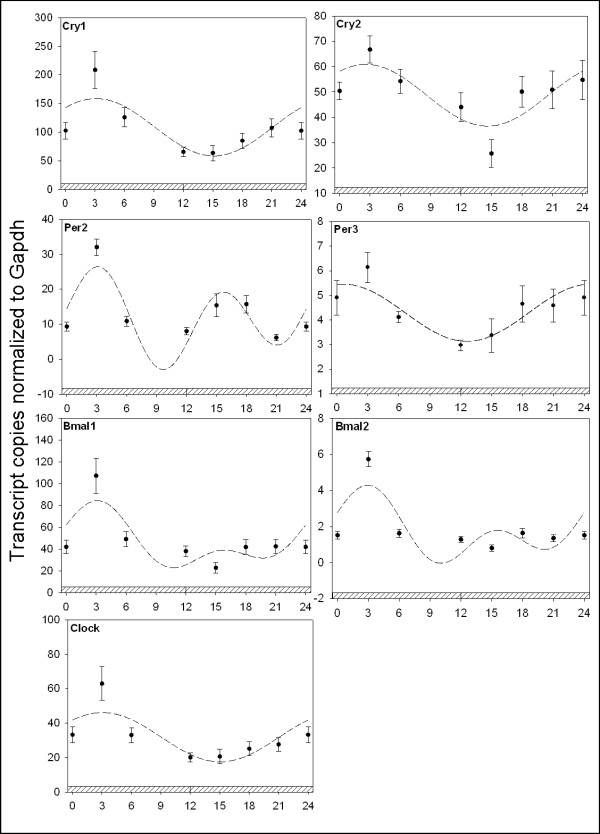
**Quantitative RTPCR analysis of circadian clock gene expression in the spleen**. Plotted dark circles represent the mean ± SEM in each experimental group. The dashed line represents the fitted plot of cosinor analysis utilizing linear harmonic regression. Values are represented as the number of transcript copies/1000 Gapdh transcripts. Abscissa labels indicate ZT time values every 3 hrs under DD conditions; crosshatched indicates darkness. Clock genes display robust 24 hr oscillations with maximal amplitudes occurring in the late subjective night and early day for *cry1 *(p_ANOVA _< .001; p_cosinor _= .001), *cry2 *(p_ANOVA _= .006; p_cosinor _= .005), *per2 *(p_ANOVA _< .001; p_cosinor _< .003), and *per3 *(p_ANOVA _= .03; p_cosinor _= .006), *bmal1 *(p_ANOVA _< .001; p_cosinor _= .003), *bmal2 *(p_ANOVA _< .001; p_cosinor _= .003), and *clock *(p_ANOVA _< .001; p_cosinor _= .002).

The known day/night rhythmic nature of circadian clock and immune genes in the avian pineal gland and retina prompted us to investigate if circadian clock gene expression in peripheral immune tissues harbors day/night rhythms [19,25]. Daily oscillations in the spleen are evident, as clock genes exhibit 24 hr oscillations in mRNA abundance (Figure 1). Examination of the putative negative elements, the *cry *and *per *genes, reveals daily oscillations with 2-5 fold amplitudes with higher abundances occurring during the late night for *cry1 *(p_ANOVA _< .001; p_cosinor _= .009), *cry2 *(p_ANOVA _= .003; p_cosinor _< .001)*, per2 *(p_ANOVA _< .001; p_cosinor _= .005), and *per3 *(p_ANOVA _< .001; p_cosinor _= .008). Analysis of the putative positive elements, *clock *and the *bmals*, also reveals a daily pattern of rhythmicity in the spleen. Clock mRNA attained maximal abundance during the early night (p_ANOVA _< .001; p_cosinor _< .001) while *bmal1 *is highest during the late night to early day period (p_ANOVA _= .007; p_cosinor _= .01). *Bmal2 *exhibited increased expression during the nighttime (p_ANOVA _= .01; p_cosinor _= .03), however it was not as robust as other clock genes nor in excess of a 2-fold rhythm. The period1 (*per1*) gene has not been identified in birds and is thus not examined here. Under free running conditions (DD), clock genes display robust 24 hr oscillations with maximal amplitudes occurring in the late subjective night and early day for *cry1 *(p_ANOVA _< .001; p_cosinor _= .001), *cry2 *(p_ANOVA _= .006; p_cosinor _= .005)*, per2 *(p_ANOVA _< .001; p_cosinor _< .003), and *per3 *(p_ANOVA _= .03; p_cosinor _= .006), *bmal1 *(p_ANOVA _< .001; p_cosinor _= .003), *bmal2 *(p_ANOVA _< .001; p_cosinor _= .003), and *clock *(p_ANOVA _< .001; p_cosinor _= .002) (Figure 2). To our knowledge this is the first demonstration of daily and circadian clock gene regulation in an avian immune tissue.

**Figure 3:**
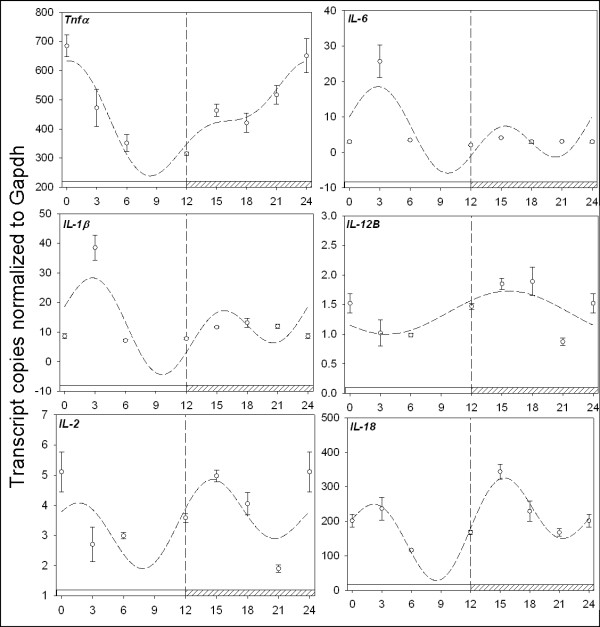
**Quantitative RTPCR analysis of cytokine gene expression in the spleen**. Plotted open circles represent the mean ± SEM in each experimental group. The dashed line represents the fitted plot of cosinor analysis utilizing linear harmonic regression. Values are represented as the number of transcript copies/1000 GAPDH transcripts. Abscissa labels indicate ZT time values every 3 hrs under LD 12:12 conditions; ZT0 = lights on; ZT12 = lights off. Open bar on the bottom of the x-axis indicates the light period, while crosshatched indicates darkness. TNFά mRNA attains maximum levels at the dark-light transition (p_ANOVA _< .001; p_cosinor _= .003), IL-1β (p_ANOVA _< .001; p_cosinor _< .001), and IL-6 (p_ANOVA _< .001; p_cosinor _< .001) mRNA each displayed a prominent peak shortly after lights on ~ZT3, while IL-18 (p_ANOVA _< .001; p_cosinor _= .003) achieved maximum values prior to midnight (ZT15). IL-2 and IL-12β mRNAs did not exhibit a >2-fold rhythm in LD, although the mRNA levels for both fluctuated during the course of the day.

**Figure 4:**
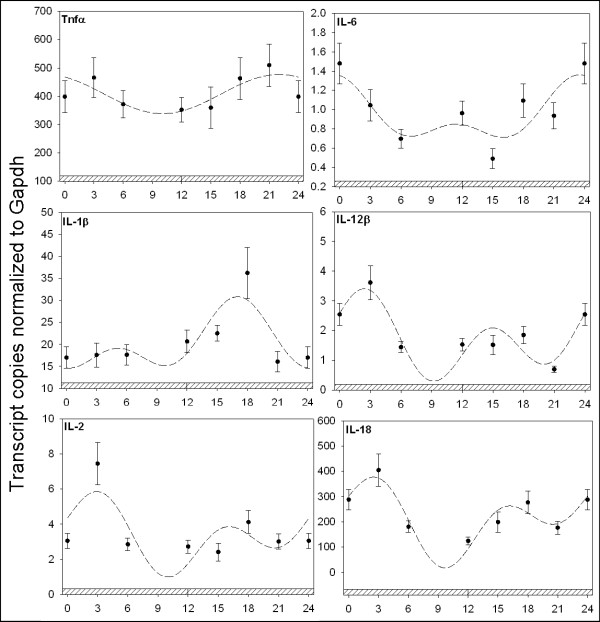
**Quantitative RTPCR analysis of cytokine gene expression in the spleen**. Plotted dark circles represent the mean ± SEM in each experimental group. The dashed line represents the fitted plot of cosinor analysis utilizing linear harmonic regression. Values are represented as the number of transcript copies/1000 GAPDH transcripts. Abscissa labels indicate ZT time values every 3 hrs under DD conditions; crosshatched indicates darkness. TNFά became statistically arrhythmic at a >2-fold change while IL-1β (p_ANOVA _= .004; p_cosinor _= .003) and IL-6 (p_ANOVA _< .001; p_cosinor _= .003) mRNAs maintained robust circadian oscillations with maximal levels occurring at subjective midnight and subjective dawn, respectively. IL-2 (p_ANOVA _< .001; p_cosinor _= .003), IL-l2β (p_ANOVA _< .001; p_cosinor _= .002) and IL-18 (p_ANOVA _< .003; p_cosinor _= .006) revealed that the mRNAs for each reached maximal values at early subjective day.

We next explored the hypothesis that components of the inflammatory pathway are regulated in a daily manner in the spleen. First, we examined the daily regulation of cytokines mobilized following insult to the host by injury or infection, including TNFά, IL-1β, IL-6 and IL-18. Understanding the regulation of these cytokines by the circadian clock represents plausible novel therapeutic opportunities for intervention during the development of autoimmune diseases [29]. Under daily conditions, the temporal mRNA levels of TNFά, IL-1β, IL-6, and IL-18 all express rhythmic profiles over a 24 hr period (Figure 3). TNFά mRNA attains maximum levels at the dark-light transition (p_ANOVA _< .001; p_cosinor _= .003), IL-1β (p_ANOVA _< .001; p_cosinor _< .001), and IL-6 (p_ANOVA _< .001; p_cosinor _< .001) mRNA each displayed a prominent peak shortly after lights on ~ZT3, while IL-18 (p_ANOVA _< .001; p_cosinor _= .003) achieved maximum values prior to midnight (ZT15). IL-2 and IL-12b mRNAs did not exhibit a >2-fold rhythm in LD, although the mRNA levels for both fluctuated during the course of the day (Figure 3). In DD, TNFά became arrhythmic (Figure 4) while IL-1β (p_ANOVA _= .004; p_cosinor _= .003) and IL-6 (p_ANOVA _< .001; p_cosinor _= .003) mRNAs maintained robust circadian oscillations with maximal levels occurring at subjective midnight and subjective dawn, respectively. The DD expression profiles for IL-2 (p_ANOVA _< .001; p_cosinor _= .003), IL-l2b (p_ANOVA _< .001; p_cosinor _= .002) and IL-18 (p_ANOVA _< .003; p_cosinor _= .006) revealed that the mRNAs for each reached maximal values at early subjective day (Figure 4).

### Daily regulation of the inflammatory response

**Figure 5:**
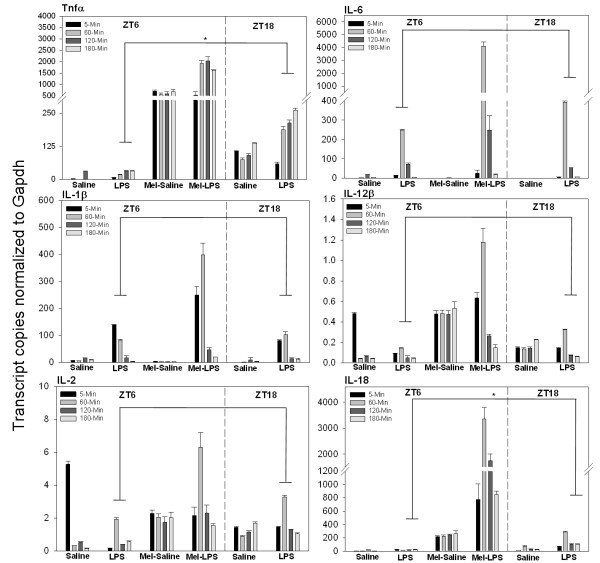
**Effects of acute melatonin and LPS administration upon cytokine induction in the spleen at midday versus midnight**. Plotted values represent the mean ± SEM in each experimental group. Values are represented as the number of transcript copies/1000 GAPDH transcripts following the respective treatments, lipopolysaccharides (LSP), melatonin (Mel), or saline (Sal), ZT6 = midday; ZT18 = midnight. Melatonin was administered one hour prior to challenge with LPS or saline (ZT5). *Statistical significance is based on *P *< 0.05.

Our experiments demonstrate that several inflammatory cytokines oscillate in a daily and circadian manner within the spleen (Figures 3, 4). We therefore hypothesized that the circadian clock functions as a regulatory mechanism of the inflammatory response. Investigation of a time-of-day-dependent inflammatory response was performed by challenging the immune system with LPS at midday or midnight and assaying proinflammatory cytokine induction. We observed that a set of cytokines displays greater overall induction during the night vs. the day, indicating daily regulation for the dynamics of the inflammatory response (Figure 5). For example, TNFά exhibits an ~8-fold greater overall induction at midnight (ZT18) versus midday (ZT6) following immune challenge (p_ANOVA _< .001). However, there is an approximately equal 2.5-fold averaged induction of TNFά levels in the three hours following LPS stimulation at both ZT6 (p_ANOVA _= .05) and ZT18 (p_ANOVA _< .001) vs. saline control. Upon closer examination, the resulting magnitude of TNFά induction at midnight is a reflection of daily TNFά regulation, as control levels at ZT18 are higher than TNFά control levels at ZT6, consistent with our temporal analysis of TNFά (Figure 4). This mechanism of daily cytokine regulation is also applicable to IL-18 induction, which exhibits a very similar response dynamic, an ~7-fold greater overall induction at midnight (ZT18) vs. midday (ZT6) following immune challenge (p_ANOVA _< .001). However, there is an approximately equal 3-fold average induction of IL-18 levels following LPS stimulation at both ZT6 (p_ANOVA _< .001) and ZT18 (p_ANOVA _< .001) vs. saline control (Figure 5). IL-2 and IL-12β mRNAs did not exhibit large average inductions following LPS stimulation at either midday or midnight, consistent with studies indicating that these cytokines function downstream of the acute inflammatory response [30].

IL-1β and IL-6 harbor more markedly different daily inflammatory response profiles. In contrast to TNFά and IL-18, there is not a large averaged induction at midnight vs. midday following LPS stimulation for either molecule. However, the magnitude of cytokine induction as a result of systemic inflammation is greater at midnight than midday for both IL-1β and IL-6 mRNA, 15-fold (p_ANOVA _< .001) vs. 6-fold (p_ANOVA _< .001) for IL-1β, and 331-fold (p_ANOVA _< .001) vs. 14-fold (p_ANOVA _< .001), respectively for IL-6 (Figure 5). The mechanism to explain such a result may again lie with the circadian clock and the rhythmic expression patterns of inflammatory cytokines. IL-1β and IL-6 both exhibit decreased levels during the night as compared to the daytime (Figure 3). Summarily, the daily dynamics of the inflammatory response for both IL-1β and IL-6 appear to be in an anti-phase relationship to that of IL-18 and TNFά dynamics in the spleen due to circadian clock control.

### Melatonin control of the inflammatory response

The rhythmic nocturnal production of the hormone melatonin has an impact upon a wide range of physiological functions throughout the body by functioning as a transducer of timing information to peripheral cells and tissues [31,32]. For this reason, it represents an attractive signaling molecule for studying mechanisms of synchronization between the central circadian clock and peripheral oscillators. We therefore investigated the effect of exogenous melatonin administration upon the daily dynamics of the inflammatory response. In the case of TNFά and IL-18, melatonin administered at midday mimicking nighttime physiological levels resulted in 70-fold (p_ANOVA _< .001) and 34-fold (p_ANOVA _< .001) increases in abundance respectively vs. control (Figure 5). Further, a 170-fold (p_ANOVA _< .001) increase in TNFά mRNA levels was elicited when melatonin was administered prior to immune stimulation (LPS). This enhanced induction effect appears to be a reflection of the combined actions of both molecules. For example, TNFά is induced 2.5-fold following LPS stimulation alone at midday, while melatonin elicits a 70-fold induction. The combined predicted induction of both molecules would be 175-fold (2.5 × 70 = 175), which is very close to our observed 170-fold increase. Similarly, IL-18 is increased an average 3-fold following LPS induction alone at ZT6 (p_ANOVA _< .001), 34-fold by melatonin (p_ANOVA _< .001,) and 217-fold (p_ANOVA _< .001) by both melatonin and LPS treatment together. Thus, an additive or synergistic affect of melatonin and LPS function is evident in the regulation of TNFά and IL-18 cytokine induction following inflammation in the spleen.

Similar to our findings in the LPS induction experiment, IL-1β and IL-6 behave in a contrasting fashion to TNFά and IL-18. IL-1β and IL-6 mRNA are decreased in abundance following melatonin administration at midday (Figure 5). However, pre-treatment with melatonin at midday caused LPS-induced cytokine levels of IL-1β and IL-6 to exhibit massive increases in abundance compared to LPS treatment alone. For example, IL-6 increases 14-fold (p_ANOVA _< .001) following LPS treatment at ZT6, decreases 11-fold (p_ANOVA _< .001) following melatonin administration, and increases 176-fold (p_ANOVA _< .001) after subsequent melatonin and LPS treatment, resulting in absolute levels 12-fold higher than LPS treatment alone. Likewise, IL-1β is increased 6-fold (p_ANOVA _< .001) following LPS treatment at midday, decreased 3-fold by melatonin at ZT6, and increased 18-fold (p_ANOVA _< .001) by both melatonin and LPS treatment together. IL-2 and IL-12β showed only slight average increases following either melatonin or melatonin/LPS administration, indicating that although expression of these cytokines is weakly stimulated by melatonin, it does not act in conjunction with LPS or enhances its effects, at least within the 3 hr time frame of the data sampling. These data reveal that melatonin decreases the expression of IL-1β and IL-6 mRNA at midday but also functions in a synergistic fashion with LPS in the induction of these cytokines during inflammation.

## Discussion

The attention paid to chronobiology and chronotherapy has grown progressively in recent years as circadian rhythms are now recognized as intertwined with several aspects of immune function, including the regulation of cytokine production, leukocyte trafficking, proliferation, and apoptosis [33-39]. However, we still do not know all the molecular pathways, structures, or timing signals responsible for communication between the circadian clock and immune system nor do we know how dysfunction adversely affects homeostasis. In the present study we provide important insight into circadian control of inflammatory mechanisms and open new avenues toward deciphering circadian control of immune tissues and their immunological rhythms.

### Circadian clock gene regulation in the spleen

Rhythmic oscillation of avian circadian clock genes has been previously demonstrated in pineal and retinal tissues [19,25,40]. These and several additional studies have further prompted the notion that molecular clocks reside not only in central oscillatory structures, but also in peripheral tissues and cells [3,41,42]. In addition, we now know that molecular clocks outside of the brain are essential regulators of normal peripheral physiology [43]. In this study we show that circadian clock genes possess daily oscillations in spleen tissue and that these oscillations persist under constant conditions indicative of regulation by the circadian clock and are not merely driven by light-dark cycles (Figures 1, 2). The temporal distributions of the mRNA for these clock proteins are interesting. First, there is no strict antiphase of positive and negative elements that has been observed in *Drosophila *and mammalian models. Here, both *bmal*s are expressed coincidentally with *crys*. This temporal pattern of clock gene mRNA abundance is similar, but not identical, to that found in the avian pineal gland [19]. In the pineal gland, the *bmals and crys*, are all expressed rhythmically with high amplitudes in the same phase, all of which were confirmed by northern analysis. We do not, at this stage, have any data concerning either the level or sub-cellular localization of the proteins that these genes encode, but the similarities in temporal distribution between the chick spleen and pineal gland, and the differences between the temporal distributions of these mRNA species in these chick tissues and those of mammals and flies strongly suggest that other rhythmic mechanisms are in place that regulate molecular rhythms among these model systems. In addition, the cellular composition of the spleen (B cells, T cells, macrophages, dendritic cells, natural killer cells) represents a highly dynamic population of cell types and functions. For example, it is possible that subpopulations of specific spleen cell types harbor their own molecular clocks and that overt spleen inflammatory function is a result of independent timing signals originating from a subpopulation of cell types. Thus, additional work is required to acquire a comprehensive understanding of clock gene expression within different cell populations of the spleen and their necessity in regulating specific daily immunological functions.

### Proinflammatory cytokine regulation in the spleen

IL-1β, IL-6, and TNFά are proinflammatory cytokines that function as key regulators of the early response to inflammation [44]. Understanding cytokine regulation under various photoperiodic conditions in the spleen and the ability of melatonin to modulate their expression under normal and inflammatory states is integral to deciphering the dynamics of the inflammatory response [45]. In this study several proinflammatory cytokines exhibited daily and circadian patterns of expression in the spleen (Figures 3, 4). This result suggests that the circadian clock is responsible for a daily rhythm of the inflammatory response due to endogenous inflammatory cytokine regulation by the circadian clock. TNFά and IL-18 both exhibit greater overall induction at midnight (ZT18) versus midday (ZT6) following immune challenge due to elevated levels for each during the night (Figure 5). However, it is not clear whether local or systemic timing signals are responsible for daily control of cytokine expression. A systemic timing mechanism to explain the nocturnal rise in abundance for both the TNFά and IL-18 cytokines may be attributed to melatonin. As previously stated, melatonin is a hormone produced by the pineal gland and its synthesis characteristically increases nocturnally. This phenomenon occurs in all vertebrate species, regardless of their being diurnal or nocturnal organisms. The magnitude and duration of the nocturnal increase in melatonin synthesis is dependent upon the length of the dark-phase of the light cycle, acting as an entrainment cue for multiple biological functions [46]. Among the multiple regulatory functions attributed to melatonin, the hormone's role in antioxidant defense and the immune system has been documented [47]. The immunomodulatory impact of melatonin has been examined using several experimental approaches with many conflicting results. In rats, studies indicate that melatonin administration exacerbates several immune parameters while pinealectomy produces an opposite effect, resulting in a reduction of immune parameters [48,49]. However, in mice, inhibition of melatonin synthesis with propanolol administration during the evening, and daily injections of p-chlorophenylalanine, resulted in a significant depression of the primary antibody response to sheep red blood cells [48]. In birds, the immunoenhancing and anti-glucocorticoid activities of melatonin were not observed in certain studies [50,51]. Therefore, although connections between the immune system and melatonin are evident, the mechanism appears quite complex and species-dependent.

In this study it is evident that while melatonin regulates the inflammatory response it is not the sole timing mechanism capable of cytokine regulation in the spleen. For example, IL-1β and IL-6 mRNA profiles exhibit peak mRNA abundances at ZT3, in opposition to TNFά and IL-18 mRNA profiles (Figure 3). Further, neither IL-1β nor IL-6 is induced following acute exogenous melatonin administration (Figure 5). These data suggest that an alternative circadian timing mechanism of cytokine regulation is present in the spleen in addition to melatonin. Participation by more than one hormone or chemical mediator, especially if each was controlled by daily light cycles differently or interacted with one another, could account for the diurnal phase differences in immune responses. In the context of this study, it is probable that melatonin does not function alone as a mediator of inflammation but rather interacts with other hormones. For example, Moore and Siopes [52] reported that the stimulatory effects of melatonin on cellular and humoral immune responses of quail were dependent on opioids. Additional studies have also shown that melatonin interacts with glucocorticoids and testosterone to affect daily changes in immune function [48,53]. In the present study, the ability of melatonin to elicit diurnal changes in inflammatory immune function in the spleen supports a primary role for melatonin in controlling daily changes in inflammatory function but does not exclude involvement of other mediators. Further study is needed in this area to identify the mechanism(s) and any additional mediators regulated by the circadian clock that are involved in modulating the immune response.

## Conclusions

Ultimately, understanding the broad role of the circadian clock and its timing cues in modulating immune function will have significant impacts on the fields on chronobiology, physiology, immunology, and endocrinology. Revealing how the circadian and immune systems are able to accomplish balanced homeostasis without detriment to the host organism will provide new insight into the role of the circadian clock and further our understanding of an organism's health.

## Methods

### Materials

Melatonin and lipopolysaccharides (LPS from Escherichia coli 0111:B4) were obtained from Sigma-Aldrich.

### Animals

One-day-old male chicks, *Gallus gallus*, Hy-line Brown, were obtained from Hyline International (Bryan, TX). For daily and circadian time series experiments involving 7 timepoints, animals were housed for 3 weeks in a LD 12:12 photoperiod with food and water continuously available (total n = 63 for the daily experiment, total n = 63 for the circadian experiment). The 7 time points tested in LD were ZT0, ZT3, ZT6, ZT12, ZT15, ZT18, and ZT21 (ZT: zeitgeber time, lights on at ZT0; lights off at ZT12). For the DD experiments animals were held in constant darkness for 3 days prior to sampling under dim red light at CT0, CT3, CT6, CT12, CT15, CT18, and CT21 (CT: circadian time). All animals were sacrificed by CO_2 _asphyxiation and tissues removed were immediately placed on solid CO_2 _and stored at -80°C until use. Three pools of tissue were prepared at each time point, each of which was composed of three spleens (n = 9 per time point). Animal use and care protocols were in accordance with NIH guidelines.

### Quantitative real-time polymerase chain reaction

Total RNA (4 μg/sample) was isolated from each pool using the Trizol protocol (Invitrogen), according to manufactures instructions. Total RNA was then subjected to DNase treatment using TURBO DNA-*free *(Ambion) to remove contaminating genomic DNA. The amount of RNA was assessed using an Eppendorf Biophotometer (Eppendorf). cDNA production was performed following the High Capacity cDNA Reverse Transcription Kit protocol (Applied Biosystems) using 1 μg of DNase-treated total RNA as starting material. qRTPCR determinations were made using a LightCycler 480 (Roche). Reactions (20 μl volume) contained 0.5 μM primers, SYBR Green mastermix (Roche), and cDNA, according to the manufacturer's instructions. All incubations included an initial denaturation step at 95°C for 10 minutes, typically followed by 40 cycles of a 95°C denaturation for 15 s, 30 s annealing at 63°C, then extension at 72°C for 30 s. Primers used for qRTPCR are described in Table 1; all primer pairs generated a single product of the predicted size, as indicated by agarose gel electrophoresis. Thereafter, specificity was demonstrated during every qRTPCR run by melting curve analysis (Tm). Typically ~25 cycles were necessary to detect amplification. All qRTPCR assays were linear (r2 > 0.99) from 10^1 ^to 10^7 ^copies.

**Table 1:** List of primers used for qRTPCR.

Target	Identity	Sequence	Accession #	Size
**Bmal1**	Fwd	ggaattccaggaggaacaaga	AF205219	60
	Rv	ttcttcagcaatcatccttcc		

Bmal2*	Fwd	gaagtccggtataaaccttcgtt	AF246958	65
	Rv	gcagccctaaggattaactgtct		

**Clock**	Fwd	acacgcatgatagaggcaaa	AF246959	62
	Rv	tgttcttgaattttccgcaact		

Cry1*	Fwd	cggacctgtacaaaaaggtaaaa	NM_204245	61
	Rv	agctggccatagagggagag		

**Cry2**	Fwd	tctggcgggagtttttctac	NM_204244	60
	Rv	cctccatgcgatcaaacttc		

**Per2**	Fwd	cgaggtcagggggttctact	NM_204262	61
	Rv	gatatcagcttgctgctcagg		

Per3*	Fwd	tttaggctctcactcctgtgaa	AY046567	60
	Rv	ttgctgtttttcccactgtct		

**IL-1β**	Fwd	ggtggccatgaccaaact	NM_204524	61
	Rv	caggtcgctgtcagcaaag		

**IL-2**	Fwd	gagtgcacccagcaaactct	NM_204153	66
	Rv	ttcagtttctttcttcagagtaacca		

IL-6*	Fwd	caggacgagatgtgcaagaa	NM_204628	64
	Rv	tgttccggacgagcatct		

**IL-12b**	Fwd	ccaccgaagtgaaggagttc	NM_213571	63
	Rv	cgtgggtcttagcagacagg		

IL-18*	Fwd	agagcatgggaaaatggttg	NM_204608	60
	Rv	ccaggaatgtctttgggaac		

**TNF**ά	Fwd	acaaaatttgcaggctgtttc	AY765397	60
	Rv	ctgaaataaacaggcacaaaagag		

GAPDH*	Fwd	ggagtccactggtgtcttcac	NM_204305	64
	Rv	cttagcaccacccttcagatg		

Fwd = forward primer; Rv = reverse primer; Size = expected amplicon size. Primer pairs that also spanned an intron are indicated by an *.

Transcript number was determined using internal standards, which were prepared by cloning target PCR products into pGEMT Easy vectors (Promega). Clone verification was performed by direct sequence analysis and also by plasmid DNA digestion followed by agarose gel electrophoresis (2.0%, w/v) for visualization of correct product sizes and staining with ethidium bromide (EtBr, 0.5 μg/ml). For each experiment, a set of 100-fold serial dilutions of each internal standard (10^1^-10^7 ^copies/2 μl) was prepared and used to generate standard curves. Transcript number was determined using a 2 μl sample of a 10-fold dilution of cDNA prepared as mentioned above; values were normalized to the number of Glyceraldehyde 3-phosphate dehydrogenase (GAPDH) copies.

### LPS-Melatonin Administration

For the midday exogenous melatonin experiment, day old male birds, Hy-line Brown (total n = 144 for the midday experiment) were housed in cages in LD 12:12 for approximately 5 weeks until their weight reached ~0.5 kg. An intramuscular injection (IM) of saline (n = 72) or melatonin (n = 72) (100 ul at 100 ug/kg) was delivered at ZT5. This dosage of melatonin mimics physiological nighttime levels in the chicken [54]. 1 hr following the initial injection, an intravenous (IV) injection of LPS (1.5 mg/kg, 100 ul) was delivered to half the animals and saline vehicle and to other half. Spleen tissues were harvested 5 minutes following the second injection and then every hour for 3 hrs (n = 9 per sampling, n = 36 per condition). A 3 hr time frame has proven to be an appropriate time course to analyze cytokine induction as a result of LPS stimulation [55]. For the midnight experiment, day old male birds, Hy-line Brown (total n = 72 for the midnight experiment) were housed in cages in LD 12:12 for approximately 5 weeks until their weight reached ~0.5 kg. An IV injection of saline (n = 36) or LPS (n = 36) (1.5 mg/kg, 100 ul) was delivered at ZT18. Spleen tissues were harvested 5 minutes following the injection and then every hour for 3 hrs (n = 9 per sampling, n = 36 per condition) as in the midday experiment.

### Statistical analysis

All times series data involving 7 times points were analyzed by analysis of variance (ANOVA) (Sigmaplot) and cosinor analysis utilizing linear harmonic regression (CircWave software) [56]. Significant differences among means were estimated using the Student-Newman-Keuls Method. Average changes in cytokine levels in the LPS and melatonin experiments were subjected to two-way ANOVA (Sigmaplot) and multiple comparisons vs. control group (Holm-Sidak method) for each time point. Statistical significance is based on *P *< 0.05.

## Authors' contributions

LM and SK contributed equally in carrying out the molecular studies, pharmacology experiments and sampling procedures. SK participated in the design of the study and performed the statistical analysis. MB conceived of the study, participated in its design and coordination and helped to draft the manuscript. All authors read and approved the final manuscript.
